# Complement activation in severely ill patients with sepsis: no relationship with inflammation and disease severity

**DOI:** 10.1186/s13054-023-04344-6

**Published:** 2023-02-16

**Authors:** Aline H. de Nooijer, Antigone Kotsaki, Eleftheria Kranidioti, Matthijs Kox, Peter Pickkers, Erik J. M. Toonen, Evangelos J. Giamarellos-Bourboulis, Mihai G. Netea

**Affiliations:** 1 Department of Internal Medicine, Radboud University Medical Center, Nijmegen, The Netherlands; 2 Department of Intensive Care Medicine, Radboud University Medical Center, Nijmegen, The Netherlands; 3 Radboud University Medical Center for Infectious Diseases, Radboud University Medical Center, Radboud University Medical Center, Nijmegen, The Netherlands; 4 Fourth Department of Internal Medicine, National and Kapodistrian University of Athens, Athens, Greece; 5 Fifth Department of Internal Medicine, Evangelismos General Hospital, Athens, Greece; 6 R&D Department, Hycult Biotechnology, Uden, The Netherlands; 7 Department of Immunology and Metabolism, Life and Medical Sciences Institute, University of Bonn, Bonn, Germany

**Keywords:** Sepsis, Inflammation, Complement, Septic shock, Mortality

## Abstract

**Background:**

Sepsis is characterized by a dysregulated immune response to infection. The complement system plays an important role in the host defence to pathogens. However, exaggerated complement activation might contribute to a hyperinflammatory state. The interplay between complement activation and inflammation in relationship with adverse outcomes in sepsis patients is unclear.

**Methods:**

Secondary analysis of complement factors in a prospective study in 209 hospitalized sepsis patients, of whom the majority presented with shock. Concentrations of complement factors C3, C3a, C3c, C5, C5a, and soluble terminal complement complex were assessed in ethylenediaminetetraacetic acid plasma samples collected within 24 h after sepsis diagnosis using enzyme-linked immunosorbent assays.

**Results:**

The concentration of complement factors in plasma of severely ill sepsis patients indicated profound activation of the complement system (all *P* < 0.01 compared to healthy controls). Spearman rank correlation tests indicated consistent relationships between the different complement factors measured, but no significant correlations were observed between the complement factors and other inflammatory biomarkers such as leukocyte numbers, C-reactive protein and ferritin concentrations, or HLA-DR expression on monocytes. The concentration of complement factors was not associated with Sequential Organ Failure Assessment score, the incidence of septic shock, and mortality rates (all *P* > 0.05) in this cohort of patients with high disease severity.

**Conclusions:**

Once an infection progresses to severe sepsis or septic shock, the complement pathway is already profoundly activated and is no longer related to a dysregulated inflammatory response, nor to clinical outcome. This implies that in this patient category with severe disease, the complement system is activated to such an extent that it no longer has predictive value for clinical outcome.

**Supplementary Information:**

The online version contains supplementary material available at 10.1186/s13054-023-04344-6.

## Background

Sepsis is defined as a life-threatening organ dysfunction caused by a dysregulated host response to infection [1]. This dysregulation may vary from a hyperinflammatory state, characterized by, e.g., elevated ferritin [2], to immunoparalysis, often identified by decreased monocytic (m)HLA-DR expression [3, 4].

The complement system plays an important role in the host response. Activation of complement via the classical, lectin, or alternative pathway induces activity of proteases that cleave C3 and C5, leading to the formation of the terminal complement complex (TCC) [5]. TCC binds to pathogenic or infected cells and induces cell lysis. By cleavage of C3 and C5, the split products C3a and C5a are formed, known as anaphylatoxins, which induce activation of innate immune responses [5, 6]. While the complement system is a key player in the early innate immune response to infection, the potent pro-inflammatory effects of complement activation might also induce collateral tissue damage. Therefore, the complement system can be viewed as a double-edged sword: It is essential in the defence to pathogens, but excessive activation could lead to adverse outcomes. However, it remains to be elucidated to what extent complement activation is associated with hyperinflammation in sepsis [2].

We aimed to investigate associations between the key molecules in the terminal complement pathway, C3, C3a, C3c, C5, C5a, and soluble TCC (sTCC), and the dysregulated immune response in sepsis patients. Furthermore, we explored to what extent these factors are related to adverse clinical outcomes.

## Methods

### Study design and participants

We performed a secondary analysis of a prospective study carried out in patients diagnosed with sepsis according to the Sepsis-3 criteria [1], caused by either community-acquired pneumonia, healthcare-associated pneumonia, ventilator-associated pneumonia, acute cholangitis, or primary bloodstream infection. The study was performed in 14 clinical sites in Greece between December 2017 and December 2019, in accordance with the applicable rules concerning the review of research ethics committees and informed consent (approval by the National Ethics Committee of Greece 78/17). The primary analysis focused on immunological endotypes [7]. Patients were classified into immunological endotypes based on serum ferritin concentration and mHLA-DR expression. Patients with serum ferritin concentrations ≥ 4420 ng/mL were classified as ‘hyperinflammation’ (HI) [2], regardless of their mHLA-DR expression. Patients with mHLA-DR expression ≤ 5000 antibodies bound per cell (Ab/cell) and ferritin concentrations < 4420 ng/mL were classified as ‘immunoparalysis’ (IP) [4]. If neither the criteria for HI nor IP were fulfilled, patients were categorized as ‘unclassified’ (UC).

### Study procedures

Ethylenediaminetetraacetic acid (EDTA) blood samples were collected within 24 h after sepsis diagnosis, and plasma concentrations of complement factors were assessed by commercially available enzyme-linked immunosorbent assay (ELISA) kits (more information provided in the Additional file 1: Methods). Clinical data and laboratory measurements were extracted from medical and laboratory files.

### Statistical analysis

Data analysis was performed using SPSS software version 25.0 (IBM) and GraphPad Prism software version 8.0 (GraphPad Software). Mann–Whitney *U* tests or Kruskal–Wallis tests were used for comparisons of continuous variables, whereas Chi-square tests were used for categorical variables. Correlations between complement factors and inflammatory parameters were assessed by Spearman rank correlation tests. A *P* value of < 0.05 (two-tailed) was considered statistically significant. Bonferroni correction for multiple testing was applied as appropriate. Associations between complement factors and clinical characteristics as well as disease severity were based on quartiles of concentrations of each complement factor using binary logistic regression.

## Results

**Fig. 1:**
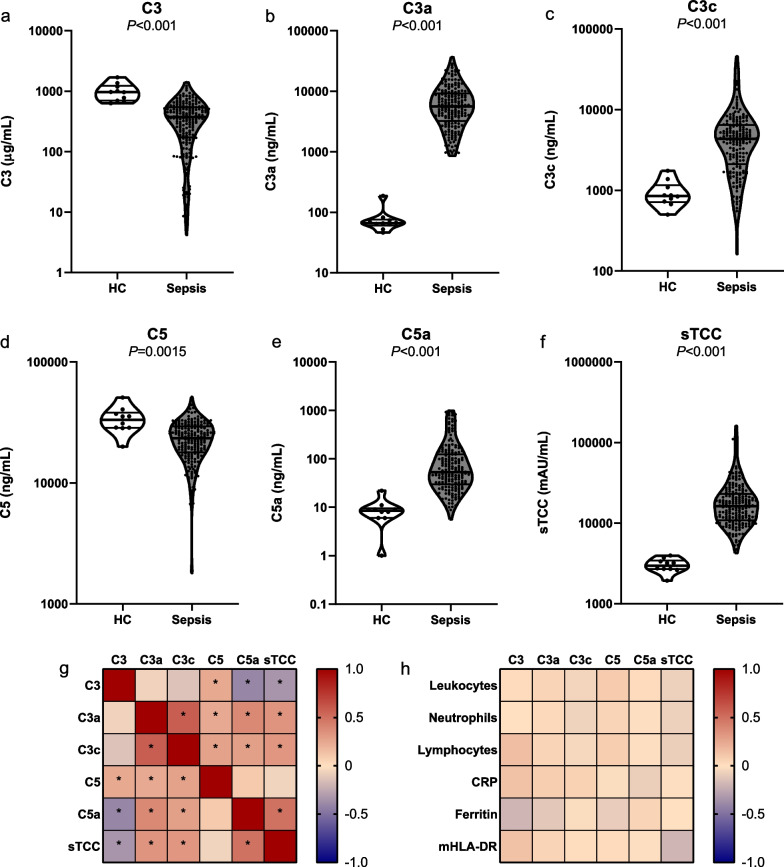
**Complement factors in patients with sepsis.** Violin plots of concentrations of the complement factors C3 (**a**), C3a (**b**), C3c (**c**), C5 (**d**), C5a (**e**), and sTCC (**f**) in plasma of patients with sepsis (*n* = 209) compared to healthy controls (*n* = 10). Data are presented as median with interquartile range on a log-scale. The *P* values in the panels were calculated with Mann–Whitney *U* tests. Heatmaps of the correlations between complement factors (**g**) and between the complement factors and inflammatory parameters (**h**). Correlation coefficients and *P* values were calculated with Spearman rank correlation tests. *Indicates *P* < 0.05 after Bonferroni correction for multiple testing. Abbreviations: HC, healthy controls; sTCC, soluble terminal complement complex; CRP, C-reactive protein

Concentrations of complement factors were measured in ETDA plasma of 209 sepsis patients and 10 healthy controls (Additional file 1: Fig. S1). The patient characteristics presented in Additional file 1: Table S1 indicate that pneumonia was the most frequent cause of sepsis and that sepsis patients were older compared to healthy controls (76 [65–84] vs. 60 [57–66], *P* < 0.001). It should be noted that the patients included in this cohort suffered from severe illness indicated by the high APACHE II score (23 [17–31]), incidence of septic shock (75%), and 28-day mortality rate (60%). Figure 1a–f shows that C3 and C5 concentrations were lower in sepsis patients compared to healthy controls (*P* < 0.001 and *P* = 0.0015, respectively), whereas C3a, C3c, C5a, and sTCC concentrations were higher in sepsis patients (all *P* < 0.001). These results imply consumption of C3 and C5 with cleavage into C3a and C3c (from C3) and C5a (from C5), finally resulting in formation of TCC. Collectively, these data indicate profound activation of the complement system in sepsis patients.

Comparisons of all complement factors revealed no significant differences between the immunological endotypes hyperinflammation (*n* = 46), unclassified (*n* = 71), and immunoparalysis (*n* = 81; Additional file 1 Fig. S2). Associations among complement factors and between complement factors and other inflammatory parameters are visualized in heatmaps (Fig. 1g–h). Although widespread correlations between different complement factors were observed, they did not correlate with leukocyte numbers, C-reactive protein (CRP), and ferritin concentrations, or mHLA-DR expression.

Based on quartiles of concentrations of the different complement factors, we assessed relationships between complement factors and clinical characteristics, as well as with the incidence of septic shock, the Sequential Organ Failure Assessment (SOFA) score and mortality (Table 1). Apart from a significant association between C3c and age (patients with higher C3c levels were younger, *P* = 0.042), no other associations with age, sex, and BMI were observed. Moreover, the SOFA score, the incidence of septic shock, and mortality rate were not associated with the plasma concentrations of any of the complement factors in this cohort of severely ill sepsis patients. Unadjusted logistic regression analyses confirmed these findings (Additional file 1: Table S2).

**Table 1:** Patient demographics and clinical outcome parameters for quartiles of complement factors

	Quartile 1 (lowest)	Quartile 2	Quartile 3	Quartile 4 (highest)	*P* value^A^
C3					
Age (years)	76 (65–83)	75 (60–83)	74 (66–85)	77 (68–86)	> 0.99
Sex, male	25 (49)	29 (57)	32 (63)	30 (59)	> 0.99
BMI (kg/m^2^)	25.8 (21.0–31.1)	26.8 (23.6–29.4)	26.3 (22.0–31.1)	26.0 (22.5–29.3)	> 0.99
SOFA	12 (9–16)	12 (10–15)	11 (8–13)	10 (8–13)	0.13
Shock	38 (75)	43 (84)	73 (75)	33 (65)	0.96
28-day mortality rate	31 (61)	38 (75)	31 (61)	24 (47)	0.27
C3a					
Age (years)	81 (67–87)	74 (65–81)	75 (67–83)	75 (62–81)	0.47
Sex, male	26 (52)	33 (66)	28 (56)	27 (54)	> 0.99
BMI (kg/m^2^)	26.1 (21.3–28.1)	26.2 (22.2–29.1)	26.3 (22.5–31.2)	27.3 (23.9–31.1)	> 0.99
SOFA	12 (10–15)	11 (7–14)	11 (8–13)	11 (9–13)	> 0.99
Shock	41 (82)	36 (72)	37 (74)	33 (66)	> 0.99
28-day mortality rate	36 (72)	32 (64)	23 (46)	28 (56)	0.32
C3c					
Age (years)	81 (68–88)	78 (70–87)	73 (63–81)	74 (64–81)	0.042
Sex, male	25 (51)	31 (63)	29 (59)	25 (52)	> 0.99
BMI (kg/m^2^)	27.8 (22.7–31.2)	24.4 (21.9–27.5)	26.1 (23.2–29.4)	27.7 (22.2–31.2)	> 0.99
SOFA	11 (9–15)	11 (8–14)	11 (8–13)	11 (6–14)	> 0.99
Shock	44 (90)	36 (74)	36 (74)	29 (60)	0.066
28-day mortality rate	34 (69)	30 (61)	31 (63)	25 (52)	> 0.99
C5					
Age (years)	77 (66–84)	75 (67–86)	77 (69–85)	73 (60–82)	> 0.99
Sex, male	33 (64)	30 (57)	30 (58)	26 (50)	> 0.99
BMI (kg/m^2^)	27.8 (24.0–31.1)	23.8 (21.3–27.7)	27.1 (24.1–29.6)	26.8 (21.6–32.1)	0.22
SOFA	11 (9–15)	11 (7–14)	11 (7–14)	11 99–13)	> 0.99
Shock	45 (87)	39 (74)	37 (71)	35 (67)	0.75
28-day mortality rate	34 (65)	28 (53)	34 (65)	30 (58)	> 0.99
C5a					
Age (years)	78 (65–86)	77 (67–86)	72 (60–79)	76 (66–84)	0.66
Sex, male	32 (65)	23 (49)	33 (69)	23 (49)	0.53
BMI (kg/m^2^)	26.1 (21.2–29.4)	27.3 (22.4–31.1)	25.8 (22.5–29.4)	27.8 (22.9–31.3)	> 0.99
SOFA	11 (10–14)	11 (7–14)	11 (8–12)	12 (9–15)	> 0.99
Shock	41 (84)	33 (70)	36 (75)	34 (72)	> 0.99
28-day mortality rate	33 (67)	27 (57)	25 (52)	32 (68)	> 0.99
sTCC					
Age (years)	81 (64–87)	76 (69–83)	76 (64–85)	73 (62–79)	0.66
Sex, male	30 (58)	31 (59)	26 (50)	32 (62)	> 0.99
BMI (kg/m^2^)	26.3 (22.8–29.8)	27.3 (22.9–29.4)	26.2 (22.7–30.7)	25.4 (20.8–33.0)	> 0.99
SOFA	11 (8–13)	11 (9–15)	11 (9–13)	11 (8–15)	> 0.99
Shock	39 (75)	39 (74)	40 (77)	38 (73)	> 0.99
28-day mortality rate	30 (58)	33 (62)	33 (64)	30 (58)	> 0.99

Data are presented as median (interquartile range) or *n* (%)

^A^*P* values are calculated with Kruskal–Wallis tests or Chi-square tests with Bonferroni correction for multiple testing

*BMI* body mass index, *SOFA* Sequential Organ Failure Assessment, *sTCC* soluble terminal complement complex

## Discussion

The results of this study point toward profound activation of the complement pathway in sepsis patients, reflected by a decrease in complement components C3 and C5 and an increase in C3a, C3c, C5a, as well as the end product sTCC. We did not observe differences in complement factor concentrations between patients with the different immunological endotypes: hyperinflammation or immunoparalysis. In line with this, no correlation with other inflammatory parameters, disease severity, or mortality was observed. Therefore, we conclude that in this cohort of sepsis patients with very severe illness, the degree of complement activation is not related to dysregulation of the immune response and clinical outcomes.

Our findings are in contrast to previous research demonstrating associations between activation of the complement pathway and mortality in sepsis patients. Studies have shown decreased circulating concentrations of C3 and C5 in non-surviving patients compared to survivors [8–10]. Subsequently, C3a and C5a concentrations were increased in non-survivors, suggesting that in these patients the complement pathway is more extensively activated [8–11]. This difference is likely to be explained by differences in timing of sample collection, sample type, pre-analytical sample handling, method of complement assessment, and disease severity between cohorts. The current study included patients based on the new Sepsis-3 criteria [1], whereas the other studies used older sepsis criteria [12, 13], hence selecting a different selection of ‘sepsis’ patients. Therefore, our results might indicate that in severely ill patients with sepsis, the complement pathway is activated to such an extent that a ceiling is reached and relationships with inflammatory or clinical parameters can no longer be identified. Since large amounts of the C3 and C5 proteins are present in the blood in homeostasis, the limiting factor(s) for additional activation of the pathway is likely upstream of these factors. However, we have only investigated the terminal complement pathway in this study, so these limiting factors remain to be elucidated. Moreover, whether this saturation of the complement pathway also leads to functional impairment of the complement system needs to be investigated.

This study has limitations that need to be addressed. First, we only measured complement factors at a single timepoint within 24 h after sepsis diagnosis. Since the complement system reacts very early in the disease course, the 24-h sampling window could already induce variation in the actual status of complement activation. We cannot exclude that repeated measurements during the disease course with strict timing intervals could provide additional insight into the relationships between complement, inflammation, and outcomes. Moreover, we cannot exclude that in sepsis patients with less severe disease, associations between complement activation and disease severity may be present. Therefore, caution is needed when extrapolating these data to other (sepsis) populations and future studies are warranted. Second, the higher age of sepsis patients compared to healthy controls could have affected our results. However, as C5 concentrations appear to increase with age [14], the already significantly lower C5 concentrations observed in sepsis patients compared to healthy controls may be an underestimation of the true difference. Therefore, this does not affect our overall conclusions. Third, as this study focused on plasma concentrations of terminal complement markers, no conclusions can be drawn regarding complement functionality or complement activity in the tissues. Fourth, due to the observational nature of this study, no causation can be inferred.

## Conclusion

Sepsis is associated with profound activation of the complement system, but in severely ill patients, circulating concentrations of complement factors are not associated with a dysregulated inflammatory response or disease severity. This may indicate that in this patient category, the complement system is activated to such an extent that it no longer has predictive value for clinical outcome.

## Supplementary Information

**Additional file 1.** Methods, Fig. S1, Table S1, Fig. S2, Table S2.

## Data Availability

The datasets used and analyzed during the current study are available from the corresponding author on reasonable request.

## Abbreviations

Ab/cell: Antibodies bound per cell
APACHE II: Acute Physiology and Chronic Health Evaluation II
BMI: Body mass index
CRP: C-reactive protein
EDTA: Ethylenediaminetetraacetic
ELISA: Enzyme-linked immunosorbent assay
HC: Healthy controls
HI: Hyperinflammation
IP: Immunoparalysis
mHLA-DR: Monocytic human leukocyte antigen—DR isotype
RCF: Relative Centrifuge Force
SOFA: Sequential Organ Failure Assessment
sTCC: Soluble terminal complement complex
UC: Unclassified
