# Supplementary material for: Complement activation in severely ill patients with sepsis: no relationship with inflammation and disease severity
Source: Crit Care. 2023 Feb 16;27:63. doi: 10.1186/s13054-023-04344-6 (PMC9933299; doi:10.1186/s13054-023-04344-6)
Supplement: Supplementary file 1 — Additional file 1. Methods, Fig. S1, Table S1, Fig. S2, Table S2. [file 13054_2023_4344_MOESM1_ESM.pdf]

Complement activation in severely ill patients with sepsis: no relationship with inflammation and disease severity

Aline H. de Nooijer[1,2,3], Antigone Kotsaki[4], Eleftheria Kranidioti[5], Matthijs Kox[2,3], Peter Pickkers[2,3], Erik J.M. Toonen[6], Evangelos J. Giamarellos-Bourboulis[4], Mihai G. Netea[1,3,7 *]

[1] Department of Internal Medicine, Radboud University Medical Center, Nijmegen, The Netherlands.

[2] Department of Intensive Care Medicine, Radboud University Medical Center, Nijmegen, the Netherlands.

[3] Radboud University Medical Center for Infectious Diseases, Radboud University Medical Center, Nijmegen, the Netherlands.

[4] Fourth Department of Internal Medicine, National and Kapodistrian University of Athens, Athens, Greece.

[5] Fifth Department of Internal Medicine, Evangelismos General Hospital, Athens, Greece.

[6] R&D Department, Hycult Biotechnology, Uden, the Netherlands.

[7] Department of Immunology and Metabolism, Life & Medical Sciences Institute, University of Bonn, Bonn, Germany.

***Corresponding author:**

Prof. Mihai G. Netea, PhD, MD

Department of Internal Medicine, Radboud University Medical Center

6500 HB Nijmegen, The Netherlands

E-mail: mihai.netea@radboudumc.nl

Methods

Study procedures

Ethylenediaminetetraacetic acid (EDTA) blood samples were collected within 24 hours after sepsis diagnosis. After collection, blood was centrifuged for 10 minutes at 3500 Relative Centrifuge Force (RCF) at room temperature and plasma was collected, aliquoted, and stored at -80°C before analysis. Concentrations of the complement factors were assessed by commercially available enzyme-linked immunosorbent assay (ELISA) kits (C3: HK366, C3a: HK354, C3c: HK368, C5: HK390, sTCC: HK328, Hycult Biotech, Uden, the Netherlands; C5a: A025, Quidel, microvue, San Diego, CA, USA) according to the manufacturer's protocols. All samples were kept on ice during pre-analytical sample handling. For each complement marker, all samples were measured at the same time, with some samples measured a second time due to results that were out of the detection range. Variance per plate was limited as checked by control samples.

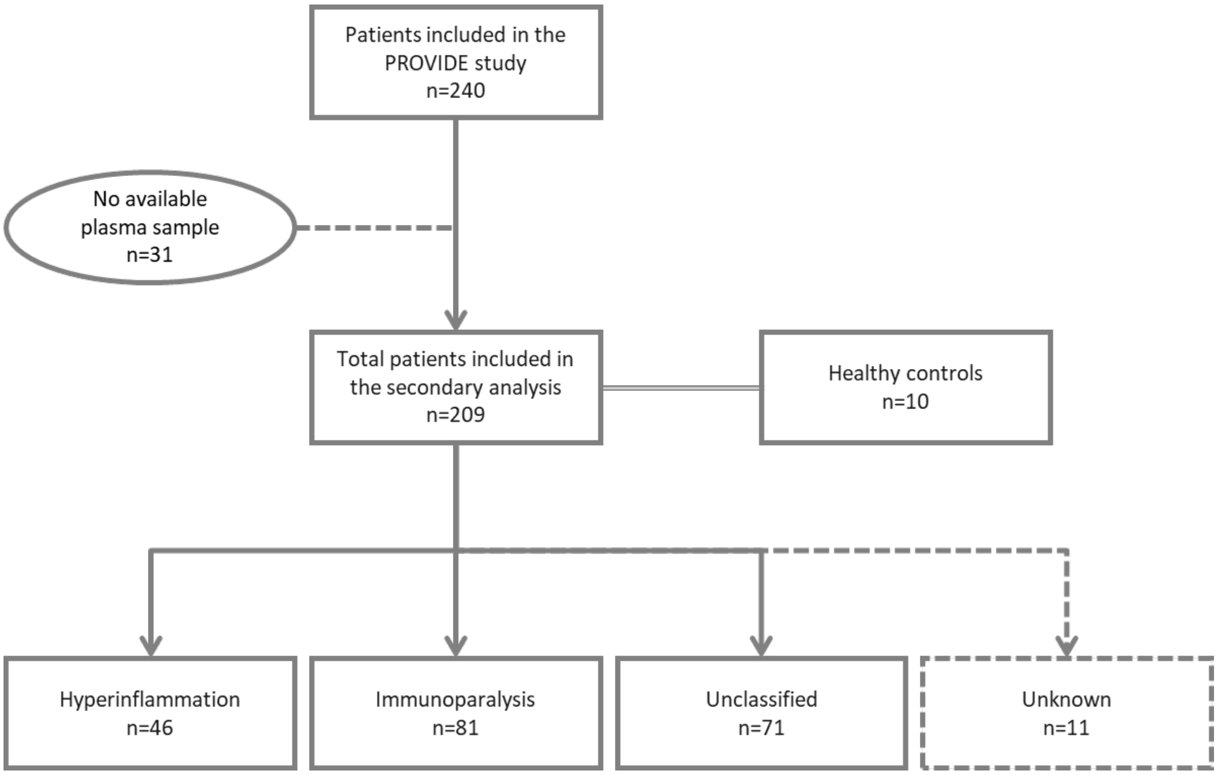

Fig. S1 Flowchart of the study.

Classification into immunological endotypes was not possible for 11 patients due to missing data on ferritin concentrations or mHLA-DR expression. EDTA plasma samples from 10 healthy controls, collected in the Netherlands, were used for comparison (the 200 Functional Genomics cohort; http://www.humanfunctionalgenomics.org).

Table S1 Patient characteristics.

	Sepsis patients (n=209)	Healthy controls (n=10)	*P*-value
**Age (years)**	76 (65-84)	60 (57-66)	**<0.001**
**Sex, no. (%)**			
Male	119 (57)	4 (40)	0.34
Female	90 (43)	6 (60)	
**BMI (kg/m^2)**	26.2 (22.5-29.8)	26.9 (23.5-29.4)	0.77
**Charlson comorbidity index**	5 (4-7)	N.A.	-
**Comorbidities**			
Diabetes mellitus	61 (29)	N.A.	-
Heart failure	52 (25)		
Coronary heart disease	43 (21)		
Chronic renal disease	26 (12)		
Chronic obstructive pulmonary disease	48 (23)		
History of malignancy	14 (7)		
**Source of infection**			
CAP	94 (45)	N.A.	-
HAP	44 (21)		
VAP	23 (11)		
Acute cholangitis	13 (6)		
Primary bacteraemia	34 (16)		
**Laboratory parameters**			
Leukocyte count (x10^9/L)	14.3 (10.0-19.8)	N.A.	-
Neutrophil count (x10^9/L)	12.0 (7.9-17.5)		
Lymphocyte count (x10^9/L)	1.0 (0.6-1.6)		
CRP (mg/L)	71 (20-176)		
Ferritin (ng/mL)	1241 (433-3219)		
mHLA-DR (Ab/cell)	4217 (2620-7270)		
**APACHE II score**	23 (17-31)	N.A.	-
**SOFA score**	11 (9-14)	N.A.	-
**Shock, no. (%)**	156 (75)	N.A.	-
**28-day mortality, no. (%)**	126 (60)	N.A.	-

Data are presented as median (interquartile range) or n (%). Abbreviations: BMI, body mass index; CAP, Community acquired pneumonia; HAP, Health care associated pneumonia; VAP, Ventilator associated pneumonia; APACHE, Acute Physiology And Chronic Health Evaluation; SOFA, Sequential Organ Failure Assessment.

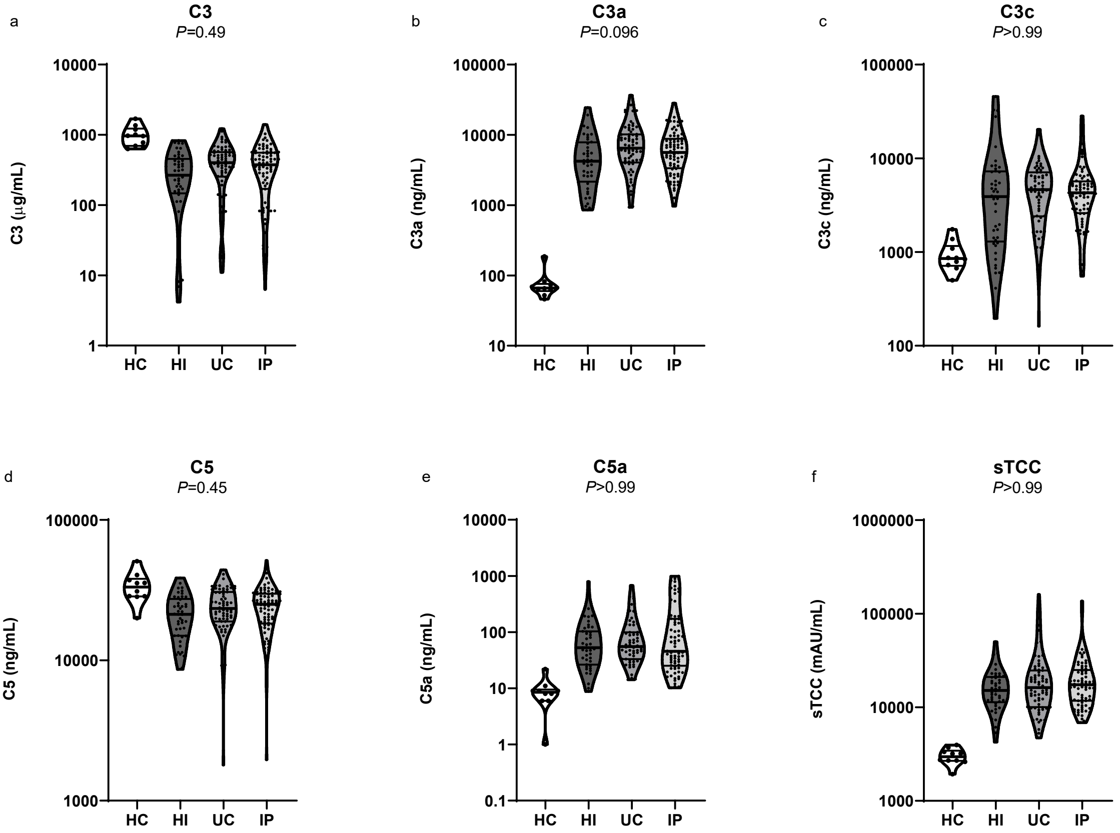

Fig. S2 Complement factors in sepsis patients with hyperinflammation, immunoparalysis and unclassified inflammatory state.

Violin plots of plasma concentrations of the complement factors C3 (a), C3a (b), C3c (c), C5 (d), C5a (e), and sTCC (f) in in HI (n=46), UC (n=71), and IP (n=81) sepsis patients. Data from healthy controls (HC) are presented as a reference. The 11 patients with unknown immunological endotype were left-out of this analysis. Data are presented as median with interquartile range. The *P*-values in the panels represent comparisons between HI, UC, and IP, calculated with Kruskal-Wallis tests. Abbreviations: HC, healthy control; HI, hyperinflammation; IP, immunoparalysis; UC, unclassified; sTCC, soluble terminal complement complex.

Table S2 Risk factors for 28-day mortality in sepsis patients.

	**Unadjusted OR**	**95%CI**	***P*-value**
Age (years)	1.038	1.017-1.060	**<0.001**
Male sex	0.677	0.384-1.192	0.18
APACHE II score	1.102	1.060-1.145	**<0.001**
C3, highest vs. lowest quartile	0.573	0.261-1.259	0.17
C3a, highest vs. lowest quartile	0.495	0.215-1.138	0.09
C3c, highest vs. lowest quartile	0.480	0.209-1.100	0.08
C5, highest vs. lowest quartile	0.722	0.327-1.596	0.42
C5a, highest vs. lowest quartile	1.034	0.439-2.434	0.94
sTCC, highest vs. lowest quartile	1.000	0.459-2.177	1.00

Odds ratios were calculated using binary logistic regression. Since no significant associations between the complement factors and 28-day mortality were observed, we did not perform multiple logistic regression.

Abbreviations: OR, odds ratio; 95%CI, 95% confidence interval; sTCC, soluble terminal complement complex.
